# Tousled-like kinase loss confers PARP inhibitor resistance in BRCA1-mutated cancers by impeding non-homologous end joining repair

**DOI:** 10.1186/s10020-025-01066-z

**Published:** 2025-01-22

**Authors:** Min-ah Kim, Banseok Kim, Jihyeon Jeon, Jonghyun Lee, Hyeji Jang, Minjae Baek, Sang-Uk Seo, Dongkwan Shin, Anindya Dutta, Kyung Yong Lee

**Affiliations:** 1 Research Institute, National Cancer Center, Goyang-Si, Gyeonggi-Do 10408 Republic of Korea; 2 Department of Microbiology, College of Medicine, The Catholic University of Korea, Seoul, 06591 Republic of Korea; 3 Department of Cancer Biomedical Science, National Cancer Center Graduate School of Cancer Science and Policy, Goyang-Si, Gyeonggi-Do 10408 Republic of Korea; 4 Department of Genetics, University of Alabama, Birmingham, AL 35233 USA

**Keywords:** DNA damage repair, Double-stranded breaks (DSBs), Non-homologous end joining (NHEJ), Homologous recombination (HR), PARP inhibitor, Tousled-like kinase, BRCA1, Cancer treatment, Cancer Therapy, Drug resistance

## Abstract

**Background:**

Double-strand breaks (DSBs) are primarily repaired through non-homologous end joining (NHEJ) and homologous recombination (HR). Given that DSBs are highly cytotoxic, PARP inhibitors (PARPi), a prominent class of anticancer drugs, are designed to target tumors with HR deficiency (HRD), such as those harboring BRCA mutations. However, many tumor cells acquire resistance to PARPi, often by restoring HR in HRD cells through the inactivation of NHEJ. Therefore, identifying novel regulators of NHEJ could provide valuable insights into the mechanisms underlying PARPi resistance.

**Methods:**

Cellular DSBs were assessed using neutral comet assays and phospho-H2AX immunoblotting. Fluorescence-based reporter assays quantified repair via NHEJ or HR. The recruitment of proteins that promote NHEJ and HR to DSBs was analyzed using immunostaining, live-cell imaging following laser-induced microirradiation, and FokI-inducible single DSB generation. Loss-of-function experiments were performed in multiple human cancer cell lines using siRNA-mediated knockdown or CRISPR-Cas9 gene knockout. Cell viability assays were conducted to evaluate resistance to PARP inhibitors. Additionally, bioinformatic analyses of public databases were performed to investigate the association between TLK expression and BRCA1 status.

**Results:**

We demonstrate that human tousled-like kinase (TLK) orthologs are essential for NHEJ-mediated repair of DSBs and for PARPi sensitivity in cells with BRCA1 mutation. TLK1 and TLK2 exhibit redundant roles in promoting NHEJ, and their deficiency results in a significant accumulation of DSBs. TLKs are required for the proper localization of 53BP1, a key factor in promoting the NHEJ pathway. Consequently, TLK deficiency induces PARPi resistance in triple-negative breast cancer (TNBC) and ovarian cancer (OVCA) cell lines with BRCA1 deficiency, as TLK deficiency in BRCA1-depleted cells, impairs 53BP1 recruitment to DSBs and reduces NHEJ efficiency, while restoring HR.

**Conclusions:**

We have identified TLK proteins as novel regulators of NHEJ repair and PARPi sensitivity in BRCA1-depleted cells, suggesting that TLK repression may represent a previously unrecognized mechanism by which BRCA1 mutant cancers acquire PARPi resistance.

**Supplementary Information:**

The online version contains supplementary material available at 10.1186/s10020-025-01066-z.

## Introduction

Double-strand breaks (DSBs) are the most cytotoxic lesions of DNA damage, and inappropriate DSB repair often leads to a premature aging-like phenotype and multiple human diseases, including tumorigenesis and genomic instability (Helleday et al. 2007; Jackson and Bartek 2009; Li et al. 2007). To repair DSBs, cells mainly exploit two pathways: non-homologous end joining (NHEJ) and homologous recombination (HR) (Ceccaldi et al. 2016). While HR occurs only in limited phases of the cell cycle (S to G2 phases) owing to the requirement of sister chromatids generated during DNA replication, NHEJ is a relatively rapid process that is activated throughout cell cycle progression (Delacote and Lopez 2008). Most DSBs are repaired by NHEJ (Mao et al. 2008).

In the early stage of HR, BRCA1-CtIP and MRN (MRE11-RAD50-NBS1) nucleases generate single-stranded DNA (ssDNA) at the end of DSBs, which is coated by the RPA complex, followed by RAD51 recombinase, which allows strand invasion to sister chromatids to copy intact DNA (Bouwman et al. 2010; Bunting et al. 2010; Chapman et al. 2013; Escribano-Diaz et al. 2013; Feng et al. 2013). The choice between NHEJ and HR at DSBs largely depends on whether the BRCA1-associated complex can access the DSB to resect the ends to create ssDNA (Daley and Sung 2014). *BRCA1* mutation, by creating HR deficiency (HRD), acts as a double-edged sword (Toh and Ngeow 2021). It increases the lifetime risk of certain types of cancer development, especially breast and ovarian cancers, but paradoxically the HRD in the tumor cells makes the cancer susceptible to therapy by poly (ADP-ribose) polymerase (PARP) inhibitors and platinum compounds. PARP inhibitors (PARPi) inhibit base excision repair (BER) and thus increase the persistence of ss breaks that are converted to DSB, which need HR for repair. PARPi has shown promising results in the treatment of breast and ovarian cancers with germline *BRCA* mutations by exploiting synthetic lethality between BER inhibition and HRD (Bryant et al. 2005; Farmer et al. 2005).

Although PARP inhibitors have shown significant benefits in terms of progression-free survival and overall response rates, not all patients with BRCA1 mutations respond to PARPi therapy. A study published in *The New England Journal of Medicine* in 2019 reported that among patients with BRCA1/2-mutated advanced breast cancer who received talazoparib, an FDA-approved PARP inhibitor, the objective response rate was 62.6% and the median progression-free survival was 8.6 months (Litton et al. 2018). However, not all patients benefited from the therapy, and the study reported that 37.4% of the patients had progressive disease. Similarly, a study published in *The Journal of Clinical Oncology* in 2020 reported that among patients with BRCA1/2-mutated ovarian cancer who received niraparib, another FDA-approved PARPi, the objective response rate was 73%, and the median progression-free survival was 12.9 months (Turner et al. 2021). However, the study also reported that 27% of the patients did not respond to therapy. Accordingly, despite the significant clinical benefits of PARPi in patients with BRCA1-mutated breast and ovarian cancers, additional research is needed to identify predictive biomarkers that can help identify patients who are likely to benefit from PARPi therapy and to develop new strategies to overcome resistance to this therapy.

Tousled-like kinases (TLKs) are a family of serine/threonine kinases conserved across a wide range of eukaryotic organisms and have been implicated in multiple cancers and developmental disorders (Segura-Bayona and Stracker 2019; Khalil and Benedetti 2022; Kim et al. 2016; Lee et al. 2018). In mammals, two homologs, TLK1 and TLK2, share a high degree of sequence similarity and are expressed throughout the cell cycle (Segura-Bayona and Stracker 2019). TLKs play a role in a variety of cellular processes, including DNA replication, chromatin remodeling, and the DNA damage response (Benedetti 2012). TLK activity is the highest during the S-phase of the cell cycle, and essential for efficient DNA replication and proper chromosome segregation (Sillje et al. 1999; Han et al. 2005). TLK phosphorylates ASF1 to promote histone chaperone activity during DNA replication (Klimovskaia et al. 2014). Although TLK is transiently inhibited by ATM-dependent Chk1 phosphorylation upon DDR (Gatei et al. 2003; Groth et al. 2003), the role of TLKs in DDR remains relatively underexplored. TLK1B, a splice variant of TLK1, regulates DNA damage checkpoints by phosphorylating Rad9 at S328, leading to dissociation of the 9-1-1 alternative clamp loader (Canfield et al. 2009). TLKs also play a role in the repair of DNA double-strand breaks, with the depletion of TLK1 leading to delayed S-phase progression and impaired DSB repair efficiency in cells recovering from irradiation (Sunavala-Dossabhoy et al. 2005). Overexpression of TLK1 has been shown to confer radioresistance in mouse mammary fibroblasts, and pharmacological inhibition of TLK with certain phenothiazines impairs DSB repair, leading to the accumulation of γH2AX (Li et al. 2001; Ronald et al. 2013). A recent study by Ghosh et al. identified a specific function for TLK1 in regulating HR repair through RAD54 phosphorylation (Ghosh et al. 2023). However, it remains unclear whether TLKs also play a role in NHEJ-mediated repair of DSBs, a critical aspect of maintaining genome stability.

In this study, we demonstrated that human TLKs play a critical role in NHEJ-mediated DSB repair and enhance PARPi sensitivity in breast and ovarian cancer cells with BRCA1 deficiency. Specifically, TLK1 and TLK2 facilitate NHEJ repair by promoting the recruitment of 53BP1 to DSBs. Furthermore, we found that depletion of either TLK1 or TLK2 in breast cancer and ovarian cancer cells with BRCA1 deficiency leads to increased resistance to PARPi. This resistance is associated with reduced NHEJ and elevated HR activity, despite BRCA1 depletion. In conclusion, the genetic status and expression levels of TLK1 and TLK2 should be investigated as potential biomarkers for predicting resistance or sensitivity to PARPi therapy in cancers with HRD.

## Methods

### Cell lines

HEK293T, U2OS, U2OS EJ5-GFP (Gunn and Stark 2012), U2OS DR-GFP (Gunn and Stark 2012), NHEJ/DsRed293B (Golding et al. 2009), U2OS ER-mCherry-LacI-FokI-DD (Shanbhag et al. 2010; Tang et al. 2013), and MDA-MB-468 cells were grown in Dulbecco’s modified Eagle’s medium (DMEM, Gibco) containing 10% fetal bovine serum (FBS, Gibco) and penicillin/streptomycin (1%, Thermo Scientific) at 37 ℃. A549 cells were cultured in RPMI 1640 (Corning) supplemented with 10% FBS and 1% penicillin/streptomycin. MDA-MB-436 cells were maintained in Leibovitz’s L15 medium (Gibco) supplemented with 10 μg/ ml insulin (12525–014, Gibco), 16 μg/ ml glutathione (G6013, Sigma-Aldrich), and 10% FBS at 37 °C in a humidified atmosphere without CO_2_. Human ovarian cancer cell line UWB1.289 cells were cultured in a mixture of 1:1 RPMI 1640 and Mammary Epithelial Growth Medium (MEGM, Lonza) supplemented with 3% FBS. U2OS EJ5-GFP and U2OS DR-GFP cells were obtained from Dr. Jeremy Stark (City of Hope), U2OS ER-mCherry-LacI-FokI-DD cells were a gift from Roger A. Greenberg (University of Pennsylvania). NHEJ/DsRed293B cells were provided by Dr. J. Larner (University of Virginia) and K Valerie (Virginia Commonwealth University).

### Knockout using the CRISPR/CAS9 system and siRNAs

Guide RNA (gRNA) targeting TLK1 or TLK2 were subcloned into lentiCRISPR v2 plasmid, which was co-transfected with two packaging vectors (psPAX2 and pMD2.G) in HEK293T cells. The culture medium was filtered with 0.45 μm syringe filter and incubated with target cells in presence of 8 μg/ml polybrene (Sigma). After 48 h, cells were selected with 2 μg/ml puromycin at least over a week. We avoid the single clonal selection to generate a heterogenous population of the knockout cells and knockout efficiency was verified by the immunoblots or immunostaining using anti-TLK1 or anti-TLK2 antibodies. The target sequences for each guide RNA are provided below. gTLK1: 5’-GATTTACTGGAGTTGCAAGT-3’, gTLK2: 5’-GAACCATATGAAACTAGCCA-3’.

Cells were transfected using Lipofectamine RNAiMAX (13778150, Invitrogen) for siRNAs according to the manufacturer’s instructions. The siRNAs were used at a final concentration of 1 or 10 nM. The sequences of the siRNAs for this study are as follows: siGL2, 5’-CGUACGCGGAAUACUUCGA-3’; siTLK1-1, 5’-GAAGCUCGGUCUAUUGUAA-3’; siTLK1-2, 5’- GCAAUGACUUGGAUUUCUA-3’; siTLK2-1, 5’-GAUAGAAAGACAACGGAAA-3’; siTLK2-2, 5’-GGAGGGAAGAAUAGAUGAU-3’; siBRCA1, 5’- CCUGUCUCCACAAAGUGUG-3’; si53BP1, 5’-GCCAGGUUCUAGAGGAUGA-3’. Unless stated otherwise, siTLK1 and siTLK2 in this paper refer to siTLK1-1 and siTLK2-1, respectively. To enhance the knockdown efficiency of TLK, siRNA transfection was performed twice at a 24 h-interval.

### Plasmids, mutagenesis and generation of stable cell lines

*TLK1* or *TLK2* sequences were PCR-amplified and cloned into the pcDNA3-HA vector. siRNA-resistant TLK1 wild-type (WT) was generated using the following primers: forward, 5’-CAAGTTAATGTCAGAGAAAGAGGCCAGGTCTATTGTAATGCAGATTG-3’ and, reverse, 5’-CAATCTGCATTACAATAGACCTGGCCTCTTTCTCTGACATTAACTTG-3. pcDNA5-FRT/TO-EGFP-53BP1 was a gift from Daniel Durocher (Addgene plasmid #60813; http://n2t.net/addgene:60813; RRID:Addgene_60813). To generate stable cell lines, the plasmids were transfected into U2OS or U2OS EJ5 cells using Lipofectamine 2000 (Invitrogen). After 48 h post-transfection, cells were treated with 500 μg/ml G418 (Invivogen) for more than a week for drug selection.

### Neutral comet assay

The neutral comet assay was performed using a CometAssay Kit (Trevigen, 4250-050-k) according to manufacturer’s instructions with minor modifications. Briefly, U2OS cells were incubated with DMSO or 3.5 μM bleomycin for 12 h, then harvested in ice-cold 1X PBS at a concentration of 1 × 10^5^ cells/mL. The cells were diluted with low-melting agarose at a 1:10 ratio and spread on a comet slide. The slides were incubated in humidity chamber for 30 min and lysed with lysis buffer (Trevigen) at 4 ℃ for 1 h. The slides were then immersed in a pre-chilled 1X TBE buffer (Enzynomics) for 15 min, electrophoresed (1 V/cm^2^) for 40 min, and washed in D.W. followed by fixation with 70% EtOH. The DNA was stained with RedSafe™ Nucleic Acid Staining Solution green (Intron, 1:20000) for 30 min at RT. All images were captured using ZEISS Axio Imager M2, and tail moment, reflecting tail length as well as the fraction of total DNA in the tail, was analyzed using cometscore2.0 software (http://rexhoover.com/index.php?id=cometscore).

### Immunoblotting and antibodies

For immunoblotting, cells were lysed by RIPA lysis buffer (GenDEPOT) containing protease inhibitors for 15 min on ice and then briefly sonicated. About 30–50 μg of lysates were loaded onto an SDS-PAGE gel and transferred to a Nitrocellulose membrane (Amersham Protran). The membranes were blocked with 3% BSA in TBS-T (0.1% Tween 20 in TBS) for 30 min at room temperature and probed with primary antibodies at 4 ℃ overnight or at room temperature for 1 h. The primary antibodies were detected with secondary antibodies at room temperature for 1 h and visualized using an ECL solution (Millipore).

The antibodies used in this study are as follows: TLK1 (4125S), γH2AX (phospho-S139) (2577S), H2AX (2595S; Cell Signaling Technology), TLK2 (sc-393506), HA (sc-7392), 53BP1 (sc-22760), BRCA1 (sc-6954; Santacruz), GAPDH (6004-1-Ig), HA (51064-2-AP), phospho-RPA32 (S4/S8) (A300-245A; Bethyl Laboratories), RAD51 (70–001; BioAcademia).

### Live cell image with microirradiation

An EGFP-53BP1-expressing plasmid was transfected into U2OS cells for 24 h. The cells were seeded on Thermo Scientific^™^ Nunc^™^ Lab-Tek^™^ II Chambered Coverglass (Thermo Fisher) with 20 μM BrdU (Sigma-Aldrich) to sensitize them to laser microirradiation. DNA double-strand breaks (DSBs) were induced by laser microirradiation (405 nm, 24%, 1 iteration, scan speed 4, zoom 5, 40 × water lens) using an LSM880 confocal microscope (ZEISS, Germany). Images were captured every 5 s, totaling 66 images, including those taken before microirradiation. The intensity of 53BP1 was quantified using Fiji software.

### Fluorescence-based reporter assays for NHEJ or HR

NHEJ and HR assays were performed using U2OS EJ5-GFP (Gunn and Stark 2012), NHEJ/DsRed293B (Golding et al. 2009), and U2OS DR-GFP (Gunn and Stark 2012) cells as previously described (Lee et al. 2017; Lee and Dutta 2021). Briefly, 3 × ${10}^{5}$ cells were seeded in 6 well plate, and 1 nM siRNAs were transfected using Lipofectamine RNAiMAX (Invitrogen). After 24 h, 1.5 or 2 μg of HA-I-SceI expression vector pCβASce was transfected using Lipofectamine 2000 (Invitrogen). Cells were harvested at 72 h post-transfection of siRNAs, and GFP or DsRed expressing cells were counted as positive cells in FL2 or FL1 channels in flow cytometric analysis (BD FACS Calibur or Verse). To apply HA-I-SceI expression to NHEJ efficiency, the amount of each protein in the HA-I-SceI and GAPDH immunoblots was quantified using ImageJ software and their ratio was normalized to the value of NHEJ efficiency.

### Immunostaining

U2OS or A549 cells were plated on glass coverslips in 6-well plates. Cells were treated with DMSO, cisplatin or bleomycin and pre-extracted with pre-extraction buffer (25 mM HEPES, pH 7.5, 50 mM NaCl, 1 mM EDTA, 3 mM MgCl_2_, 300 mM sucrose, and 0.5% Triton X-100) for 5 min on ice. For 53BP1 focus formation at the FokI site, U2OS ER-mCherry-LacI-FokI-DD cells were treated with 2 μM 4-OHT and 1 mM Shield-1 for 6 h. Cells were fixed with 4% paraformaldehyde (PC2031-050–00, Biosesang) for 10 min. After washing three times with PBST (0.1% Triton X-100 in PBS), the plates were blocked with a solution containing 10% FBS in PBST for at least 30 min at room temperature. Primary antibodies were added and incubated with the cells for 2 h, followed by detection using secondary antibodies labeled with Alexa-555 (A21429; Life Technologies) or Alexa-488 (A11029; Life Technologies) for 40 min. Intracellular nuclei were stained with VECTASHIELD^®^ Antifade Mounting Medium with DAPI (H-1200-10, Vector LABORATORIES). Images were captured using a microscope Axio Imager. M2 (Zeiss) and adjusted using ZEN 3.4 (Zeiss, blue edition) and photoshop 7.0 (Adobe).

### Cell titer-glo cell survival assay

The Cell Titer 96 AQueous One Solution Cell Proliferation Assay kit (G3581, Promega) was employed to measure cell viability according to manufacturer’s instructions. In Brief, MDA-MB-468, MDA-MB-436, and UWB1.289 cells were seeded in 6 cm dishes and transfected with siRNAs. Subsequently, 1500–2000 cells per well were plated in a 96-well plate 24 h post-transfection. MDA-MB-468, MDA-MB-436, and UWB1.289 cells were treated with olaparib (S1060, Selleckchem), or niraparib (S2741, Selleckchem) or veliparib (S1004, Selleckchem) for 144 to 168 h. A mixture of Cell Titer 96 AQueous Reagent and Media was added to the plates at a 1:5 ratio, and readings were taken using BioTek PowerWave HT 340 microplate reader. The surviving fraction was normalized to the value of the untreated controls.

### DepMap analysis

Depmap database version 23Q4 was downloaded directly from the DepMap portal (https://depmap.org/portal/). Using the metadata provided by DepMap, cell lines originating from Breast and their mutation status to BRCA1 were identified. Score type ‘effect’ was used to evaluate the dependency effect of PARP1 on these cell lines. The TLK1 and TLK2 expressions and the DepMap scores were compared between non-mutated BRCA1 cell lines and cell lines with mutations in BRCA1. Statistical significance between the two groups was assessed using the Wilcoxon test. Scatterplots were generated to ascertain the correlation between the expression of TLK1/TLK2 and the DepMap score of PARP1.

### Pan-cancer survival analysis

All TCGA data were downloaded through TCGAbiolinks package in R, and prepared according to the package’s manual. BRCA1 was considered ‘mutated’ if: a) it is recorded containing at least one single nucleotide variation (SNV), b) it has a copy number less than 2, c) its expression z-score is below 2. Moreover, for BRCA and OVCA, patients with germline mutation to BRCA1 were also considered mutated (Shah et al. 2022). Each cancer was initially divided into two cohorts based on the mutation status of BRCA1. Further analysis was conducted only when both cohorts contained more than 20 cases. Each cohort was then divided into two groups based on the expression of the TLKs, using the threshold that yielded the lowest p-value from the log-rank test of Kaplan-Meyer analysis, with a minimum sample number 10. Nine cancers met the aforementioned criteria: BLCA (Bladder Urothelial Carcinoma), BRCA (Breast Invasive Carcinoma), CESC (Cervical Squamous Cell Carcinoma and Endocervical Adenocarcinoma), COAD (Colon Adenocarcinoma), HNSC (Head and Neck Squamous Cell Carcinoma), KIRC (Kidney Renal Clear Cell Carcinoma), LUAD (Lung Adenocarcinoma), OV (Ovarian Serous Cystadenocarcinoma), and UCEC (Uterine Corpus Endometrial Carcinoma). Cox proportional hazards regression analysis was performed to calculate the regression coefficient, representing the risk likelihood of the low expression group compared to high expression group. Positive numbers indicated higher risk, while negative numbers indicated lower risk. All data analysis was carried out using R version 4.2.1. The correlation between TLK1/TLK2 and BRCA1 expression was calculated from TCGA log-normalized TPM expression data. Only the primary tumor samples were selected for correlation analysis. Pearson correlation coefficient and its associated p-values were used to evaluate its significance.

### Quantification and statistical analysis

For the statistical analysis with a p-value, the Student’s t-test was employed for the reporter assays, and one-way or two-way ANOVA tests were used for the other assays. *, **, *** and **** represent p-values of < 0.05, < 0.01, < 0.005 and < 0.0005, respectively. All data were derived from at least triplicates, and a p-value of < 0.05 was considered statistically significant.

## Results

### TLK1 and TLK2 are essential for NHEJ-mediated repair.

**Fig. 1:**
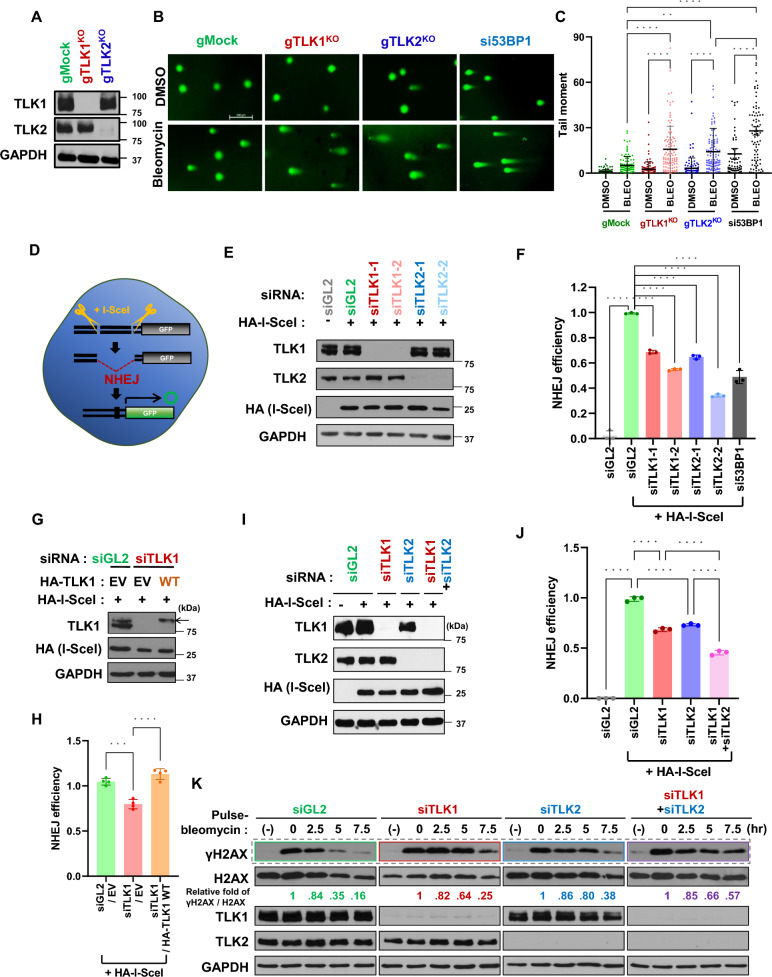
TLK1 and TLK2 promote NHEJ-mediated DSB repair. **A to C** Increased levels of DSBs following bleomycin treatment in TLK1 or TLK2 knockout (KO) cells. TLK1 knockout (gTLK1^KO^) and TLK2 knockout (gTLK2^KO^) U2OS cells were generated using the CRISPR/Cas9 system **(A)**, and a neutral comet assay was performed on the cells in presence of DMSO or bleomycin for 12 h. Representative images **(B)** and quantifications **(C)** are shown. Each dot depicts the tail moment of individual cells, and the mean with S.D. is presented from at least 100 cells. si53BP1 used as a positive control. **P < 0.01; ****P < 0.0001. Scale bar, 200 μm. **D to F** Decreased NHEJ in TLK1- and TLK2-depleted cells. Schematic of the GFP reporter assay used to measure NHEJ efficiency using U2OS EJ5-GFP cells **(D)**. Cells were transfected with two different siRNAs targeting either TLK1 or TLK2. Cell extracts were used for immunoblotting as shown in **(E)** and NHEJ efficiency was measured and plotted in **(F)**. Transient expression of HA-I-SceI was observed in the immunoblot using anti-HA antibody. The ratio of HA-I-SceI to GAPDH was quantified from the immunoblots and normalized to the percentage of GFP-positive cells in each sample. NHEJ efficiency was represented as the relative ratio compared to the siGL2 + HA-I-SceI. si53BP1 used as a positive control. Mean ± S.D. of triplicates. ****P < 0.0001. **G and H** Restoration of NHEJ by siRNA-resistant wild type TLK1 (WT) in endogenous TLK1-depleted cells. siTLK1-1 were used, and immunoblots **(G)** and quantification of NHEJ efficiency **(H)** are shown. Mean ± S.D. of quadruplicates. EV, empty vector. ***P < 0.001; ****P < 0.0001. **I and J** Further decrease of NHEJ in co-depletion of TLK1 and TLK2. Cells were transfected with siRNAs against TLK1 and/or TLK2. Immunoblots **(I)** and NHEJ efficiency **(J)** are shown. Mean ± S.D. of triplicates. **K** Delayed disappearance of bleomycin-induced γH2AX (phospho-H2AX at Ser139) in TLK-depleted cells. After transfection with the indicated siRNA, U2OS cells were pulse-treated with 20 μg/mL bleomycin for 1 h and harvested at the indicated time points. The amount of protein in the γH2AX and H2AX immunoblots was quantified using ImageJ software, and their ratio was calculated

Human TLK orthologues, TLK1 and TLK2, are critical for the maintenance of genomic integrity by participating in multiple genetic processes (Segura-Bayona and Stracker 2019). However it is unknown whether there is a distinct role between TLK1 and TLK2 in DNA damage repair. Therefore, we generated TLK1 or TLK2 knockout cells using the CRISPR/Cas9 system. The knockout of each TLK did not affect the protein level of the other (Fig. 1A). Using these cell lines, we measured the repair efficiency of DSBs, induced by a radiomimic agent bleomycin in the neutral comet assay (Fig. 1B and C). The unrepaired DSBs were quantified as the tail moment in this assay as observed in the positive control using si53BP1, and we found that both TLK1 (gTLK1) and TLK2 (gTLK2) knockout cells exhibited a significant increase in tail moment compared to control cells (gMock) (Fig. 1B, C and S1). This result indicates that TLK’s function on DSB repair is not restricted in specific orthologues, that is, both are independently playing essential roles in DSB repair. Since several high-throughput screening studies suggested that TLKs are potential candidates physically interacting with NHEJ regulatory proteins such as RIF1 and LC8, we determined to examine functional relationship between TLKs and NHEJ-mediated DSB repair. Therefore, we performed an NHEJ reporter assay using U2OS EJ5-GFP cell line (Gunn and Stark 2012) and analyzed the effect of TLK deficiency on the NHEJ efficiency. In this cell line, intact GFP can be generated when the I-SceI cut site on a mutant GFP gene is successfully repaired by NHEJ in the cells (Fig. 1D). We used two different siRNAs for each TLK to show that the effect of each depletion did not decrease the other, and that two independent siRNAs produced similar deficits in NHEJ (Fig. 1E and F). Also, the NHEJ efficiency was diminished by TLK depletion without any decrease in I-SceI expression, a marker of DSB cut on the GFP cassette (Fig. 1E). The diminished NHEJ observed upon TLK1 depletion was successfully restored by siRNA-resistant version of wild-type TLK1, confirming that the reduction in NHEJ was not due to the off-target activity of the siRNAs (Fig. 1G and H). Interestingly, while depletion of either TLK1 or TLK2 decreased NHEJ efficiency, co-depletion of both proteins caused more pronounce decrease compared to single knockdowns, indicating that TLK1 and TLK2 have redundant roles in promoting NHEJ-mediated repair of DSBs (Fig. 1I and J). The kinetics of NHEJ-mediated DSB repair can also be monitored by the time-dependent disappearance of γH2AX (phospho-H2AX at Ser-139) following bleomycin treatment. (Fig. 1K). TLK1 or TLK2 depletion delayed γH2AX disappearance by approximately 2.5 h, and this delay was further extended when both proteins were co-depleted, confirming that TLK1 and TLK2 are independently required for efficient NHEJ repair. Together, these data strongly suggest that TLK1 and TLK2 play redundant but independent roles in promoting NHEJ-mediated DSB repair.

### TLK loss impairs efficient DSB localization of 53BP1

**Fig. 2:**
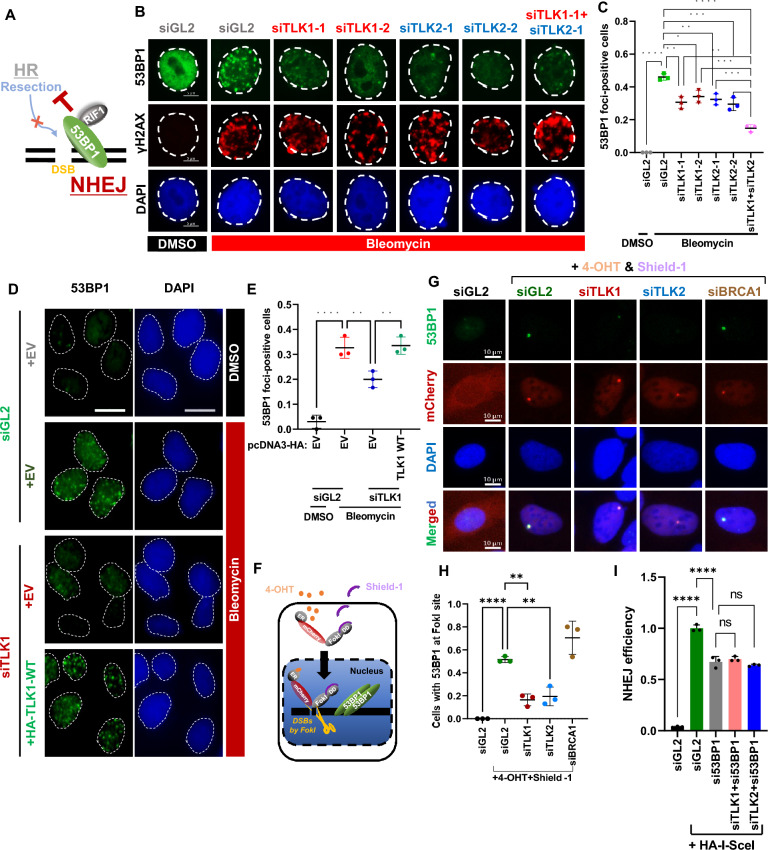
TLK loss impairs DSB localization of 53BP1. **A** A schematic explaining the critical role of 53BP1 localization at DSBs in promoting NHEJ by antagonizing DSB end resection. **B and C** Suppression of 53BP1 focus formation at DSBs in TLK-depleted cells. A549 cells transfected with the indicated siRNAs were treated with 10 μg/mL bleomycin for 1.5 h. Representative images are shown in **(B)**, and cell lysates were subjected to immunoblotting (Fig. S2A). Cells with > 20 foci were counted as positives and plotted in **(C)**. Hereafter, siTLK1 and siTLK2 indicate siTLK1-1 and siTLK2-1, respectively. Mean ± S.D. of triplicates. *P < 0.05; **P < 0.01; ***P < 0.001; ****P < 0.0001. **D and E** Restoration of 53BP1 focus formation by siRNA-resistant TLK1 in endogenous TLK1-depleted cells. A549 cells stably expressing EV or siTLK1-resistant TLK1 was subjected to siRNA transfection to deplete endogenous TLK1. Representative images **(D)** and quantification of 53BP1 foci-positives **(E)**. Scale bar, 10 μm. **F** A schematic of U2OS ER-mCherry-LacI-FokI-DD cells to monitor the recruitment of protein-of-interest at artificial DSBs generated by the FokI endonuclease. The ER-mCherry-LacI-FokI-DD fusion proteins is stabilized and translocated into the nucleus by co-treatment with Shield-1 and 4-OHT to generate DSBs at LacO repeats. **G and H** Reduced localization of 53BP1 at the FokI cut site in TLK1- or TLK2-depleted cells. U2OS ER-mCherry-LacI-FokI-DD cells were transfected with the indicated siRNAs and stained with an anti-53BP1 antibody. Cells were counted when 53BP1 foci colocalized with mCherry. Representative images are shown in **(G)** and the counted % graphed in **(H)**. **I** TLK’s NHEJ function depends on 53BP1. The NHEJ assay using U2OS EJ5-GFP cells was performed with the indicated siRNAs, as described in Fig. 1D to F. Data are presented as mean ± S.D. of triplicates. ****P < 0.0001; ns, not significant

In the cellular choice of repair pathway between NHEJ and HR at DSBs, the localization of 53BP1 to DSBs promotes NHEJ by antagonizing the formation of ssDNA coated with RPA and Rad51 at DSBs (Xu and Xu 2020) (Fig. 2A). To investigate this further, we examined the localization of the NHEJ-promoting factor 53BP1 in TLK-depleted cells (Fig. 2B and C). A549 cells were depleted of TLK1 and/or TLK2, and stained with an anti-53BP1 antibody to observe bleomycin-induced 53BP1 foci at DSBs. We observed that γH2AX was unchanged upon TLK depletion, indicating that TLK depletion does not affect the formation of DSBs or the early response to DNA damage (Fig. 2B and S2A). As expected, the number of 53BP1 foci-positive cells decreased in TLK1- or TLK2-depleted cells, and co-depletion led a further statistically significant reduction (Fig. 2B and C). The decrease in 53BP1 foci-positive cells were successfully rescued by re-expression of HA-TLK1, confirming that the reduction in 53BP1 foci is specifically due to the loss of TLK and not off-target effects (Fig. 2D and E). To further validate these results, we introduced a single DSB at a defined site using U2OS ER-mCherry-FokI-DD cells treated with Shield-1 and 4-OHT. The Shield-1 binds to the degron to stabilize the FokI while the 4-OHT binds to the ER fused to FokI, allowing nuclear translocation of the protein (Fig. 2F). Treatment of 4-OHT and Shield-1 in the cells enabled mCherry-linked-FokI nuclease to bind to specific FokI cut sites, and the mCherry signal allowed visualization of the localization of the protein of interest at the FokI cut. Depletion of TLK1 or TLK2 significantly diminished the intensity of 53BP1 signal at the FokI site, while depletion of BRCA1, used as a negative control, did not show any reduction compared to si-control (Fig. 2G and H). Additionally, we performed laser-induced microirradiation to measure the kinetics of EGFP-53BP1 recruitment to laser-induced DSB lesions in live cells (Fig. S2B and S2C). In control cells, EGFP-53BP1 was efficiently enriched at laser-induced DSB stripes, whereas TLK knockout cells exhibited impaired recruitment. We also confirmed that bleomycin-induced 53BP1 focus formation was suppressed in TLK1- or TLK2-knockout cells (Fig. S2D and S2E). However, the subtle defects in DSB localization observed in these TLK knockout cells suggest the possibility of TLK deficiency indirectly influencing 53BP1 DSB localization cannot be completely ruled out. Therefore, we aimed to confirm whether TLK’s regulation of NHEJ occurs within the 53BP1-mediated pathway by investigating the additive effects of TLK depletion on NHEJ in 53BP1-depleted cells. Notably, we observed no additional reduction in NHEJ beyond that caused by 53BP1 depletion alone when TLK was co-depleted. This finding suggests that TLK functions within the same pathway as 53BP1 to regulate DSB repair (Fig. 2I and S2F). Taken together, across multiple experimental systems, we conclude that TLK1 and TLK2 are essential for the proper localization of 53BP1 at DSB sites.

### TLK loss confers PARPi resistance in *BRCA1* mutated-breast and ovarian cancer cells.

**Fig. 3:**
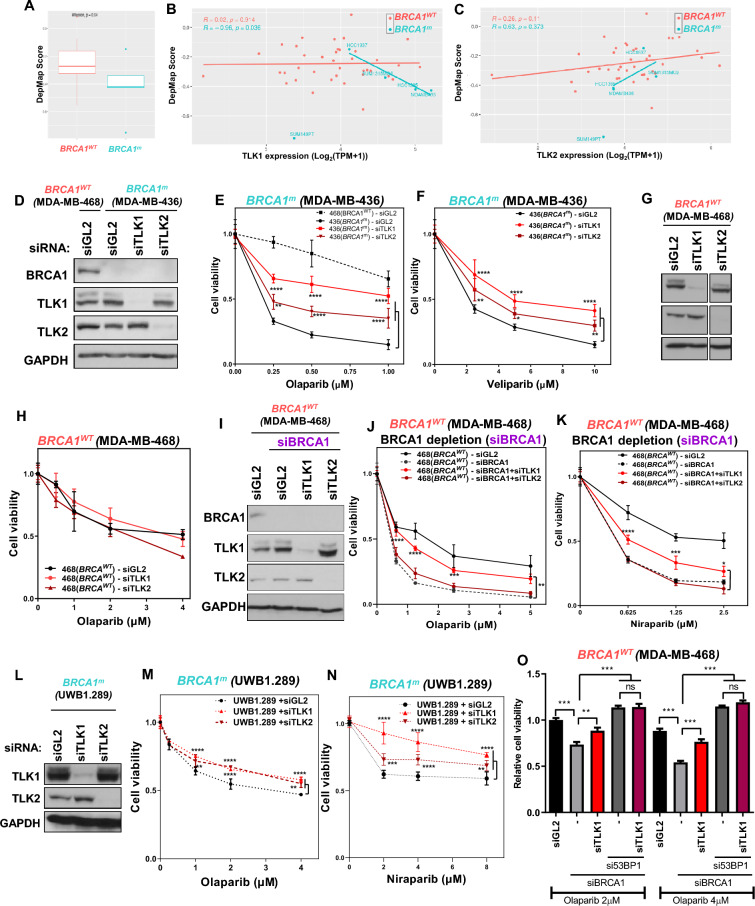
TLK deficiency increases PARP inhibitor resistance in BRCA1-mutated cancers. **A** Dependency effect of PARP1 on BRCA1 mutation status using the DepMap breast cancer cell line dataset. DepMap scores for cells with wild type and mutated BRCA1 were compared, and statistical significance was assessed using the Wilcoxon test. The DepMap score reflects the impact of PARP1 knockout on cell survival. **B and C** Correlation analysis of TLK1 **(B)** or TLK2 **(C)** expression with PARP1 DepMap scores using breast cancer cell lines from the CCLE database. DepMap scores were compared between cell lines with wild type and mutated BRCA1. **D to F** Increase of PARP inhibitor resistance following TLK1 or TLK2 depletion in BRCA1-mutated MDA-MB-436 breast cancer cells. After siRNA transfection, cell viability was measured five days post-treatment with PARPi at indicated concentration. Immunoblots **(D)** and cell survival quantification following olaparib **(E)** and veliparib **(F)** treatment are shown. Data are presented as mean ± S.D. of triplicates. *P < 0.05; **P < 0.01; ****P < 0.0005. **G and H** Effect of TLK loss on PARP inhibitor sensitivity in BRCA1-proficient MDA-MB-468 cells. Immunoblots for the indicated siRNAs **(G)** and quantification of cell survival **(H)** are shown. **I to K** Enhanced PARP inhibitor resistance following TLK1 depletion in siBRCA1-transfected MDA-MB-468 cells. Immunoblots **(I)**, cell survival quantification following olaparib **(J)** and niraparib **(K)** treatment are shown. **L to N** Increased PARP inhibitor resistance in depletion of TLK1 or TLK2 in BRCA1-mutated UWB1.289 HGSOC cells. Immunoblots **(L)**, cell survival quantification following olaparib **(M)** and niraparib **(N)** treatment are shown. **P < 0.01; ****P < 0.0005. **O** PARPi resistance caused by 53BP1 depletion in BRCA1-deficient cells is not strengthened by TLK1 co-depletion. MDA-MB-468 cells were treated with the indicated siRNAs and concentrations of olaparib, and the cell viability of all samples was normalized to that of siGL2 treated with 2 μM olaparib. ns, not significant

PARP inhibitor (PARPi) specifically targets HR deficiencies, such as BRCA1 mutations, in tumor cells, and loss of NHEJ renders BRCA1 mutant cells resistant to PARPi (Jackson and Moldovan 2022). Intriguingly, loss of 53BP1, observed in 20% of PARPi-resistant breast cancer PDXs, mechanistically restores HR by allowing the access of DNA nucleases (Cruz et al. 2018; Waks et al. 2020). To investigate the role of TLK expression in modulating cell death following PARP inhibition or deficiency in BRCA1-deficient cancers, we categorized breast cancer cell lines from the CCLE based on the presence or absence of BRCA1 mutations. We then assessed the dependency of cell death on PARP1 knockout using the DepMap database. In this analysis, the DepMap score quantifies the effect of PARP1 knockout on cell viability, where lower scores indicate an increased tendency of cell death upon PARP1 loss. Despite the limitation of having only five BRCA1 mutant cell lines available in CCLE breast cancer panel, we found that these BRCA1-mutant cell lines, out of 43, exhibited significantly lower DepMap scores compared to BRCA1 wild-type cell lines. This suggests that the DepMap scores accurately reflect the sensitivity of BRCA1-mutant cells to the anticancer effects of PARP disruption (Fig. 3A). Interestingly, we observed that TLK1 expression was inversely correlated with the DepMap score in BRCA1-mutant cells, with one exception (SUM-149PT) (Fig. 3B). These results indicate that cell death induced by PARP1 knockout in BRCA1-mutant cells tends to increase with higher TLK1 expression, while this correlation is absent in BRCA1 wild-type cells (Fig. 3B). Notably, TLK2 expression did not exhibit the same correlation with the DepMap score (Fig. 3C), suggesting that TLK1 and TLK2 may have differential effects on cellular sensitivity to PARP disruption in the context of BRCA1 deficiency, and that these effects may be cancer type- and context-dependent.

We then tested whether TLK loss could confer PARPi resistance. MDA-MB-436, a triple negative breast cancer (TNBC) cell line with a natural *BRCA1* mutation and the highest expression of TLK1 among the CCLE cell lines (Fig. 3B), exhibited significantly higher sensitivity to olaparib than MDA-MB-468, a BRCA1-proficient TNBC cell line, in a cell survival assay (Fig. 3D and E). Depletion of TLK1 or TLK2 in MDA-MB-436 cells led to increased resistance to the PARP inhibitors olaparib and veliparib (Fig. 3E and F). Interestingly, TLK1 depletion conferred greater PARPi resistance than TLK2 depletion, consistent with the DepMap analysis showing a specific correlation between TLK1 expression and PARP dependency (Fig. 3B). In contrast, depletion of TLK1 or TLK2 in BRCA1-proficient MDA-MB-468 cells did not alter PARPi sensitivity in the cell survival assay using olaparib (Fig. 3G and H). However, BRCA1 depletion in BRCA1-proficient MDA-MB-468 cells sensitized them to olaparib and niraparib, while co-depletion of TLK1 reversed this sensitivity and rendered the cells resistant to these PARPi (Fig. 3I to K). Similarly, in UWB1.289, a BRCA1-mutant high-grade serous ovarian cancer (HGSOC) cell line, TLK1 or TLK2 depletion decreased sensitivity to olaparib and niraparib (Fig. 3L to N). Since TLK1 deficiency impairs the localization of 53BP1 to DSBs (Fig. 2), and loss of 53BP1 increases PARPi resistance in BRCA1-deficient cell lines (Jaspers et al. 2013), we next investigated the role of 53BP1 in TLK1 deficiency-induced PARPi resistance in the context of BRCA1 deficiency. MDA-MB-468 cells were used to compare PARPi resistance in BRCA1 deficiency with 53BP1 single depletion and co-depletion of 53BP1 and TLK1 (Fig. 3O). The increased sensitivity to olaparib observed in BRCA1 depletion was dramatically reversed to resistance by si53BP1; however, additional depletion of TLK1 did not further enhance the resistance (Fig. 3O). These results indicate that the PARPi resistance induced by TLK1 deficiency in BRCA1-deficient cells is mediated through 53BP1. Overall, our data suggest that TLK expression is functionally linked to PARPi sensitivity in BRCA1-mutant cells and highlight the role of TLK proteins in modulating the anticancer effects of PARP inhibition, particularly in challenging cancer types such as TNBC and HGSOC.

### TLK loss restores HR in BRCA1 deficient cells

**Fig. 4:**
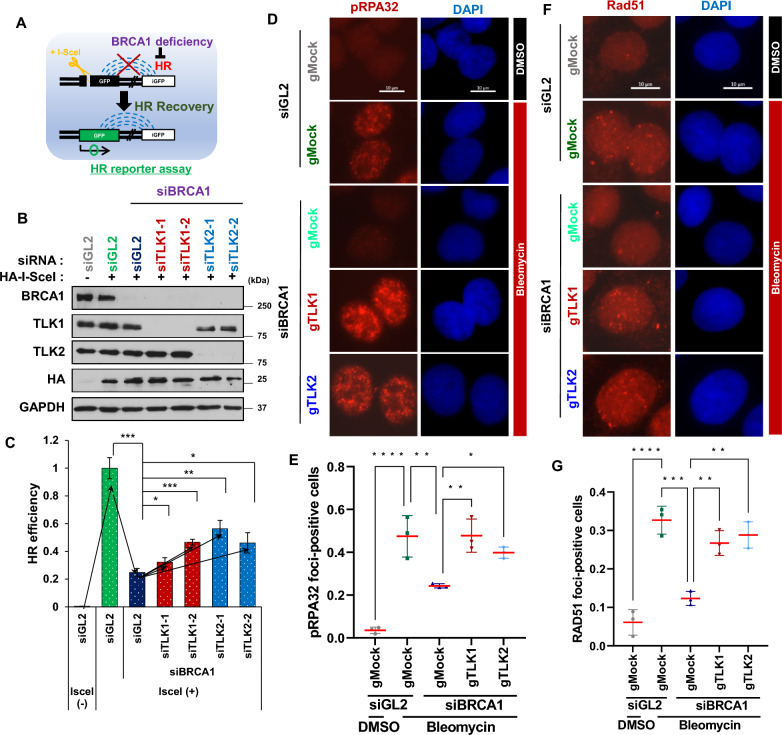
TLK loss restores HR in BRCA1-deficient cells. **A** Schematic depicting the measurement of HR repair efficiency using U2OS DR-GFP cells. Cells were transfected with siBRCA1 to reduce HR efficiency, and recovery of HR was measured by GFP reporter expression via FACS analysis. **B and C** Restoration of HR efficiency following TLK depletion under BRCA1 deficiency. Immunoblots of cell extracts **(B)** and HR efficiency **(C)** after depletion of the indicated proteins are shown. Mean ± S.D. of triplicates. *P < 0.05; **P < 0.01; ***P < 0.005. **D to G** Restoration of nuclear pRPA32 and RAD51 foci following TLK1 or TLK2 knockout in BRCA1-deficient cells. BRCA1 was depleted by siRNA in the indicated cell lines. Cells were treated with DMSO or bleomycin for 2 h, and pre-extracted before fixation. Cells with more than 20 and 5 foci were counted as positive for pRPA32 and RAD51, respectively. Representative images **(D and F)** and quantification of foci-positive cells **(E and G)** for nuclear pRPA32 and Rad51 are shown. Mean ± S.D. of triplicates. *P < 0.05; **P < 0.01; ***P < 0.005; ****P < 0.0005. Scale bar, 10 μm

BRCA1 is essential for HR, and since HR deficiency promotes the toxicity of PARPi, the effect of PARPi is maximized in BRCA1-mutated, HR-deficient cancers (Lord et al. 2015). However, inactivation of NHEJ in HRD allows the nucleases required for HR to partially restore HR, which is an important mechanism for acquired resistance to PARPi (Jaspers et al. 2013; Li et al. 2020). Because we showed that TLK deficiency promotes resistance to PARPi, we investigated whether this resistance was due to the restoration of HR in the BRCA1 deficient cells when TLK is lost. We used the U2OS DR-GFP cell line to access HR efficiency (Fig. 4A) (Gunn and Stark 2012). HR efficiency decreased to 25% upon BRCA1 depletion. However, co-depletion of TLK1 or TLK2 in BRCA1-depleted cells restored a significant portion of HR, with HR efficiency reaching approximately 55% of wild-type cells (Fig. 4B and C). This HR rescue was not due to an increase in BRCA1 protein levels upon TLK1 or TLK2 co-depletion (Fig. 4B). Similarly, pRPA32 (phosphorylated RPA32 at S4/S8) and RAD51 foci, which were reduced by BRCA1 depletion, were restored in TLK1 or TLK2 knockout cells (Fig. 4D to G and S3). Since pRPA and RAD51 are robust markers for efficient end resection during HR (Cruz et al. 2018; Sartori et al. 2007; Yamane et al. 2013), these results indicate that the defect in HR repair in BRCA1-deficient cells is rescued by TLK1 or TLK2 depletion. Although it cannot be entirely ruled out that TLK deficiency might have subtly contributed to HR restoration through changes in cell cycle progression, given that HR and its regulatory factors are highly dependent on the cell cycle, the cumulative results clearly indicate that TLKs are essential for maintaining the suppression of HR in BRCA1-deficient cells.

### TLK deficiency in BRCA1 deficient cells causes defects in 53BP1-mediated NHEJ at DSBs

**Fig. 5:**
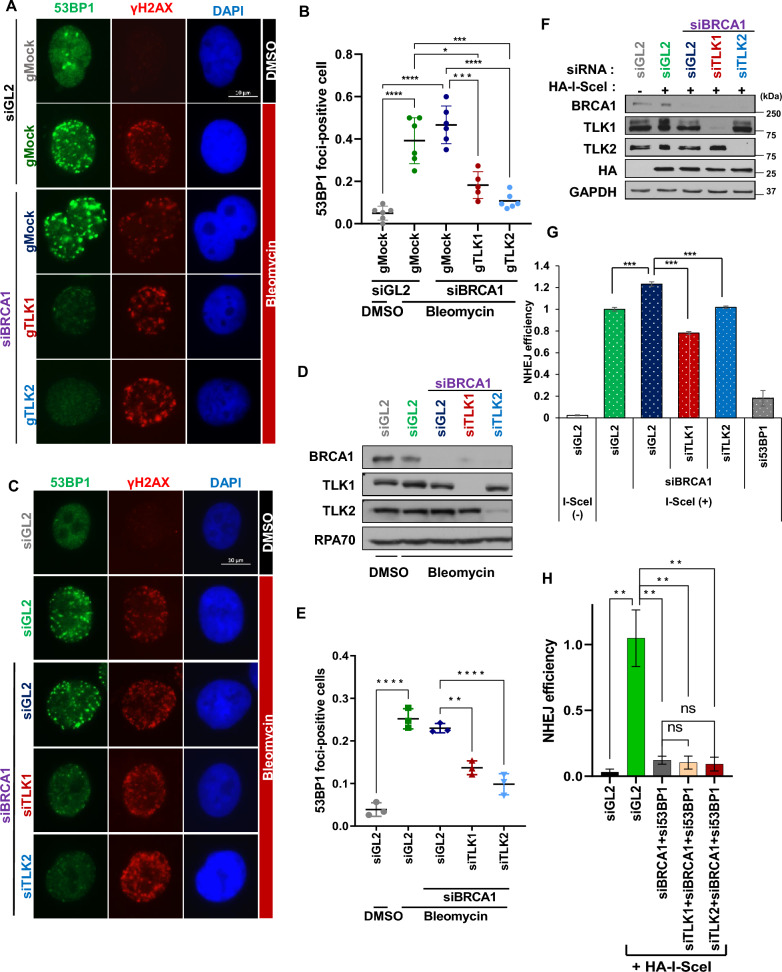
TLKs are essential for 53BP1-mediated NHEJ in BRCA1-deficient cells. **A** and **B** TLK1 or TLK2 knockout suppresses 53BP1 focus formation at DSBs in the absence of BRCA1. U2OS TLK knockout cells were transfected with si-control (siGL2) or siBRCA1 for 48 h, and treated with bleomycin before fixation. Representative images **(A)** and quantification of 53BP1 foci-positive cells **(B)** are shown. *P < 0.05; ***P < 0.005; ****P < 0.0005. Scale bar, 10 μm.** C to E** Reduced 53BP1 foci at DSBs following TLK1 or TLK2 depletion in BRCA1-deficient A549 cells. 53BP1 foci-positive cells are quantified after indicated siRNA transfections. Representative images **(C)**, immunoblots **(D)**, and quantification of 53BP1 positives **(E)** are shown. **, P < 0.01; ****, P < 0.0005. **F and G** Decrease of NHEJ efficiency by TLK depletion in BRCA1-deficient cells. NHEJ/DsRed293B cells were transfected with siBRCA1 and the indicated siRNAs. Immunoblots **(F)** and FACS analysis of DsRed-positive cells **(G)** are shown to assess NHEJ efficiency. si53BP1 used as a positive control. Mean ± S.D. of triplicates. ***P < 0.005. **H** 53BP1-dependent role of TLK in NHEJ under BRCA1 deficiency. NHEJ/DsRed293B cells were transfected with the indicated siRNAs and used to measure NHEJ efficiency. Mean ± S.D. of triplicates. **P < 0.01; ns, not significant

Next, we examined whether HR restoration upon TLK loss was due to NHEJ impairment caused by the failure of 53BP1 to localize to DSBs. Indeed, using TLK1 and TLK2 knockout cells, we observed that the number of 53BP1 foci-positive cells, which was not reduced by BRCA1 depletion, was significantly diminished in TLK1 or TLK2 knockout cells (Fig. 5A and B). Furthermore, transient depletion of TLK1 and TLK2 also resulted in a marked reduction of 53BP1 foci-positive cells, even though BRCA1 depletion did not reduce their numbers (Fig. 5C to E). These findings indicate that TLK is essential for 53BP1 binding to DSBs, even in BRCA1-deficient cells. Additionally, the reduction in 53BP1 foci-positive cells after TLK1 and TLK2 co-depletion was consistent regardless of BRCA1 status, indicating that TLK’s role in 53BP1 binding to DSBs is independent of the cell’s HR capacity (Fig. S4A and S4B). Finally, to determine whether TLK regulates NHEJ efficiency through 53BP1 under BRCA1-deficient conditions, we examined whether 53BP1 and TLK depletion exhibit an additive effect on reducing NHEJ under these conditions (Fig. 5H). We found that under co-depletion of BRCA1 and 53BP1, NHEJ was already reduced, and no further reduction was observed with the addition of siTLK1 or siTLK2. These findings indicate that the decrease in NHEJ observed following TLK depletion in BRCA1-deficient cells is dependent on 53BP1.

### TLK loss predicts good outcome in *BRCA1* wild type but not *BRCA1*-deficient tumors independent of whether the patients were treated with PARPi.

**Fig. 6:**
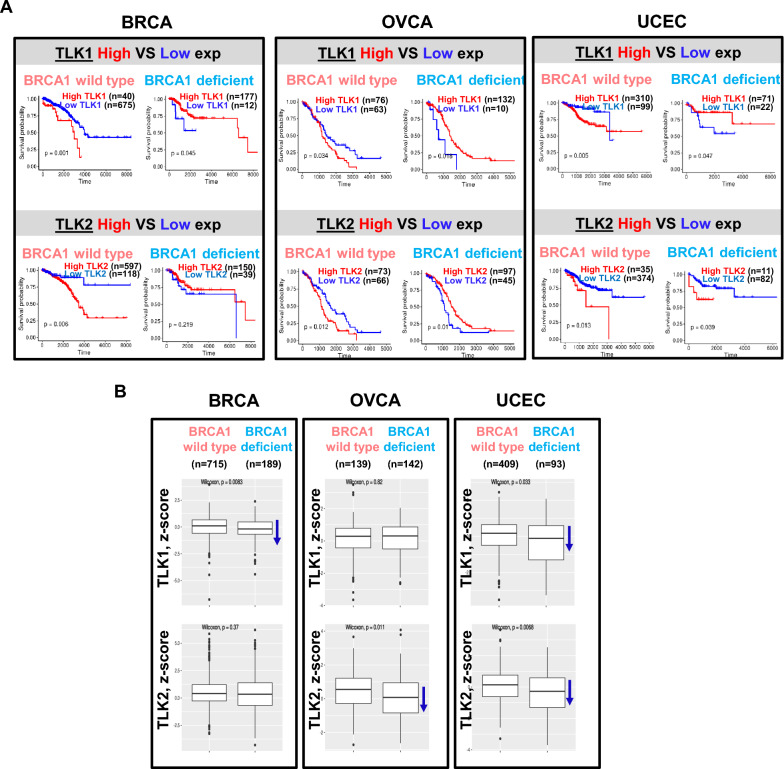
Loss of TLK correlates with poor prognosis in BRCA1-deficient patients. **A** Kaplan–Meier survival plots comparing patient groups with high and low TLK1 or TLK2 expression. Breast invasive carcinoma (BRCA), ovarian serous cystadenocarcinoma (OVCA) and uterine corpus endometrial carcinoma (UCEC) were selected in the TCGA database and divided based on the corresponding TLK expression in BRCA1 WT or deficient patients. **B** Boxplots showing TLK1 or TLK2 expression (z-score) in BRCA1 wild type and mutant cohorts. TCGA data were analyzed; n for each cohort is shown

We demonstrated that TLK loss renders BRCA1-deficient cells resistant to PARPi. Some studies have shown that TLK is elevated in certain cancers with a poor prognosis (Kim et al. 2016; Lee et al. 2018; Lin et al. 2019). In other words, TLK loss predicts a better prognosis, contrary to our observation that TLK loss makes cancers PARPi resistant. Of course, the survival studies on TLK were on patients that were not treated with PARPi. However, due to the interaction of BRCA1 status and TLK levels that we described, we hypothesized that the survival effects of TLK levels may differ in BRCA1-proficient versus BRCA1-deficient tumors, even when PARPi was not administered. To test this, we analyzed tumors in the TCGA database to investigate whether the correlation between TLK expression and patient survival is affected by BRCA1 status. Due to the insufficient number of BRCA1-deficient patients in some cancers, we analyzed nine cancers (HNSC, COAD, KIRC, UCEC, OVCA, BRCA, CESC, BLCA, and LUAD) and separated them into two groups based on BRCA1 deficiency according to our criteria (Methods). Next, each group was further divided into high and low TLK1 or TLK2 expression groups. Patients with BRCA1-proficient tumors in BRCA, OVCA, and UCEC showed poor outcomes when TLK1 or TLK2 levels were elevated, whereas patients with BRCA1-deficient tumors had better outcomes when TLK1 or TLK2 were elevated (Fig. 6A). In a broader analysis across multiple cancers, high expression of TLK1 or TLK2 was significantly associated with poor prognosis in most of cancer types with wild-type BRCA1 (or low TLK1 associated with good outcome), but this was not seen in tumors with BRCA1 deficiency (Fig. S5).

Another interesting point to note is that TLK1 expression was significantly lower in BRCA1-deficient BRCA and UCEC, while TLK2 was lower in BRCA1-deficient OVCA and UCEC, compared to their BRCA1-proficient counterparts (Fig. 6B). This result suggests that in BRCA1-deficient tumors, there may be selective pressure for decreased TLK expression even before treatment with PARP inhibitors. In other words, low TLK levels may support oncogenesis in BRCA1-deficient tumors, independent of PARPi treatment.

## Discussion

**Fig. 7:**
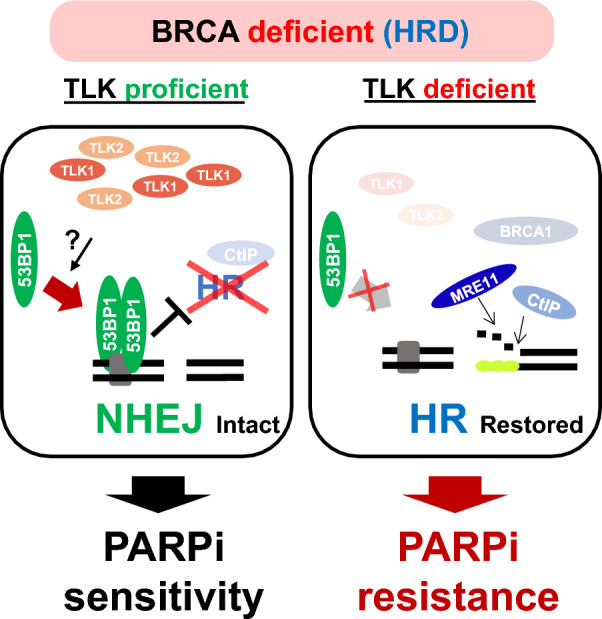
Proposed model for the role of TLK in NHEJ and PARPi sensitivity under BRCA mutation. Details are provided in the Discussion section

TLK was initially identified as an essential factor for plant development and also found to be implicated in the repair of UV-induced DNA damage (Roe et al. 1993; Wang et al. 2007). In humans, the paralogs TLK1 and TLK2 share similar substrates and play redundant roles in maintaining genome integrity (Segura-Bayona and Stracker 2019; Benedetti 2012). In this study, we identified a novel role for TLK1 and TLK2 in enhancing 53BP1 localization at DSBs, a key factor in promoting NHEJ during DSB repair. Notably, this function is not specific to a single TLK; rather, it demonstrates redundancy between TLK1 and TLK2, underscoring the essentiality of both proteins. This role is particularly critical in BRCA1-mutated cells, where HR is already compromised. We found that TLK loss induces resistance to PARPi in TNBC and ovarian cancer cells harboring BRCA1 mutations or deficiencies. Specifically, the loss of TLK further impairs 53BP1-mediated NHEJ, consequently restoring HR and conferring resistance to PARPi in these cells (as depicted in Fig. 7).

The cytotoxic effect of PARPi is diminished by NHEJ inactivation, which facilitates the restoration of HR (Li et al. 2020). Many studies have shown that the loss of 53BP1, a NHEJ-promoting factor, limits PARPi sensitivity in BRCA1 deficient conditions (Bunting et al. 2010; Jaspers et al. 2013; Li et al. 2020). Thus, the recruitment of 53BP1 to DSBs is crucial for the efficacy of PARPi, as it prevents the restoration of HR under HRD conditions. Our discovery that TLK enhances the localization of 53BP1 at DSBs, thereby inhibiting HR restoration in BRCA1 deficiency elucidate why TLK loss compromises NHEJ and renders BRCA1-deficient cells resistant to PARPi.

PARP inhibition has emerged as a promising therapeutic approach for patients with *BRCA1/2*-mutated tumors. However, acquired or de novo resistance poses a major challenge for the clinical successfulness of PARPi. Therefore, identifying new biomarkers for PARPi resistance could improve patient outcomes by informing treatment approaches. Our results suggest that low expression of TLKs may serve as a useful biomarker for predicting PARPi resistance. Additionally, cancers that are initially sensitive to PARPi may develop resistance by downregulating TLK gene expression through genetic or epigenetic changes.

Interestingly, TCGA data analysis on cancers that were not treated with PARPi suggests that a decrease in TLK kinases predicts poor outcome in BRCA1 deficient tumors, while it often predicts the opposite in BRCA1 proficient cancers. This may indicate BRCA1 deficient cancer cells benefit from a decrease in TLK even when the cells are not receiving PARPi. Consistent with this, TCGA data analysis also shows that *TLK1* or *TLK2* was decreased in expression in *BRCA1* deficient female cancers, such as BRCA, OVCA and UCEC, as opposed to those with wild type *BRCA1* (Fig. 6B) even before they are treated with PARPi. The patients who have decreased TLK expression in tumors that have not been treated with PARPi, are likely to exhibit less response to PARPi therapy, and this may explain why about one-third of patients with BRCA and OVCA with *BRCA1/2* mutation do not respond to PARPi therapy (Litton et al. 2018; Turner et al. 2021). Importantly, our research highlights that stabilizing or increasing cellular TLK expression could be a promising therapeutic approach to counteract PARPi resistance. Future studies will explore whether overexpression of exogenous TLK can indeed impair the oncogenic phenotypes of BRCA1-deficient tumors, even without PARPi treatment, and investigate the underlying mechanisms.

It has been reported that high expression of TLK1 and TLK2 is associated with poor prognosis in multiple cancers (Kim et al. 2016; Lee et al. 2018). As described above, our TCGA data analysis reveals an important distinction dependent on BRCA1 status: high expression of TLK1 and TLK2 was associated with poor prognosis in BRCA1 proficient tumors, but high expression of TLK1 and TLK2 was associated with better prognosis in BRCA1 deficient tumors. Notably, the opposing cellular sensitivity to DSBs between BRCA1-normal and -deficient cells is also observed in the loss of an antagonist of BRCA1, 53BP1 (Bouwman et al. 2010; Jaspers et al. 2013; Cuella-Martin et al. 2016). 53BP1 loss renders BRCA1-deficient cells resistant to DSB-inducing agents including platinum compounds and PARPi. This is further reinforced by our demonstration that TLK plays a role in promoting the formation of 53BP1 foci, regardless of BRCA1 status (Fig. 2B and C for BRCA1 proficiency, and Fig. 5A to E for BRCA1 deficiency).

TLK1 and TLK2 are highly conserved and share redundant roles, but they also have distinct functions. For instance, TLK2 plays a critical role in mouse placental development, whereas TLK1 is dispensable in this context (Segura-Bayona et al. 2017). In our study, we observed that the impairment of 53BP1 foci formation and HR efficiency were more pronounced in TLK2-depleted BRCA1-deficient cells compared to TLK1-depleted cells (Figs. 4 and 5). PARPi resistance upon TLK2 loss was marginal or nearly absent in TNBC cell lines (Fig. 3). As expression of TLK1 and TLK2 exhibited significantly different patterns across cancer types, this emphasizes the need to consider the distinct genetic statuses and activities of TLK1 and TLK2 based on the cancer subtype for their application in therapeutic strategies.

Future research should aim to elucidate the precise mechanisms by which TLK regulates 53BP1 activity and NHEJ. Both 53BP1 and TLK activities are dependent on and directly influenced by cell cycle progression (Groth et al. 2003; Belal et al. 2024). Since it has already been established that TLK1 knockout or knockdown does not induce cell cycle arrest (Lee et al. 2018), the defects in DSB recruitment of several DSB repair factors, including 53BP1, observed in TLK-deficient cells are not solely due to changes in the cell cycle. However, it is important to note that the same paper reported that TLK2 deficiency leads to cell cycle progression issues and DNA replication defects, raising the possibility that the changes in 53BP1 DSB localization, along with the HR factor changes observed in TLK-deficient cells, may be partially associated with changes in the cell cycle. Therefore, the cell cycle effects of each specific TLK isoform should be considered in the future, along with variations in cell lines or experimental conditions. Furthermore, it is necessary to investigate more precisely how TLK kinase activity is regulated by the cell cycle and DDR, and whether TLK influences post-translational modifications of factors regulating DSB repair. Although TLK's kinase function is transiently suppressed during the DNA damage response, it rapidly recovers to assist in DSB repair (Groth et al. 2003; Ghosh et al. 2023; Krause et al. 2003; Sunavala-Dossabhoy and Benedetti 2009). Recent work suggests that TLK1 promotes HR by phosphorylating RAD54, emphasizing the need for further exploration of TLK-mediated phosphorylation events related to 53BP1 localization at DSBs (Ghosh et al. 2023). Notably, proteins such as RIF1 and LC8, identified as TLK binding partners, are known key mediators of 53BP1 and NHEJ (Segura-Bayona et al. 2017; Mortuza et al. 2018). Investigating their interactions with TLK could provide crucial insights into the functional relationship between TLK and 53BP1-mediated NHEJ.

Our findings suggest that TLKs play a pivotal role in regulating DSB repair through NHEJ by facilitating 53BP1 localization at DSBs. This function is especially crucial in BRCA1-mutated cells, where HR is already impaired, highlighting TLK as a novel regulator of PARPi activity, a promising cancer therapy that targets HR deficiencies. Notably, the downregulation of TLK may be exploited by cancer cells as a mechanism of resistance to PARPi, even in the context of BRCA1 mutation. Furthermore, our findings indicate that the genetic status and protein levels of TLK could serve as potential biomarkers for resistance or sensitivity to PARPi therapy in BRCA1-mutated cancer cells.

## Supplementary Information

Additional file 1: Fig. S1. Related to Fig. 1B and 1C. Validation of 53BP1 depletion by si53BP1. Cells were transfected with siRNA against 53BP1 and incubated with bleomycin before harvest. Fig. S2. Related to Fig. 2. (A) Immunoblots for Fig. 2B and 2C. Cells were transfected with indicated siRNAs, and the lysates were subjected to immunoblotting. (B and C) Decreased recruitment of EGFP-53BP1 at DSBs induced by laser microirradiation in TLK knockout cells. The indicated U2OS cells were transfected with EGFP-53BP1 and irradiated with a 405 nm diode laser 24 hr post-transfection in the presence of BrdU. Live cell images (B) were captured every 5 seconds and data (C) represent the mean ± S.E.M. from eight cells. ***, P<0.001. (D and E) Decrease of 53BP1 focus formation in A549 TLK1 or TLK2 knockout cells. Representative images (D) and quantification of 53BP1 positive cells (E). Mean ± S.D. of triplicates. *, P < 0.05; **, P < 0.01: ****, P<0.0001. (F) Immunoblots for Fig. 2I. Fig. S3. Related to Fig. 4D and 4E. Immunoblots for Fig. 4D and 4E. Fig. S4. Related to Fig. 5. (A and B) Decrease of 53BP1 focus formation at DSBs in co-depletion of TLK1 and TLK2 in the absence of BRCA1. A549 cells were treated with cisplatin. Representative images (A) and quantitation (B). Scale bar, 10 μm. Mean ± S.D. of triplicates. ***, P < 0.005; ****, P < 0.0005. Fig. S5. Pan-cancer hazard analysis of low TLK1/2 cohort (Related to Fig. 6A). Cox coefficients of low TLK1/TLK2 cohorts were analyzed in multiple cancers. Red indicates higher risk (Poor outcome) and blue indicates lower risk (Good outcome) with respect to cohorts with low expression of TLK1 or TLK2. Samples are first divided by the mutation status of BRCA1, then further divided by the gene expression. ns, not significant.

## Data Availability

No datasets were generated or analysed during the current study.

## Abbreviations

DSBs: Double-strand breaks
NHEJ: Non-homologous end joining
HR: Homologous recombination
MRN: MRE11-RAD50-NBS1
ssDNA: Single-stranded DNA
BRCA1: Breast cancer gene 1
HRD: Homologous recombination deficiency
PARP: Poly (ADP-ribose) polymerase
PARPi: PARP inhibitors
TLKs: Tousled-like kinases
DDR: DNA damage response
γH2AX: Phosphorylated H2AX (a marker of DNA damage)
CRISPR: Clustered regularly interspaced short palindromic repeats
siRNA: Small interfering RNA
TNBC: Triple negative breast cancer
HGSOC: High-grade serous ovarian cancer
CCLE: Cancer cell line encyclopedia
DepMap: Dependency map
TCGA: The cancer genome atlas
UCEC: Uterine corpus endometrial carcinoma
OVCA: Ovarian cancer
BRCA: Breast cancer
