# Supplementary material for: Tousled-like kinase loss confers PARP inhibitor resistance in BRCA1-mutated cancers by impeding non-homologous end joining repair
Source: Mol Med. 2025 Jan 22;31:18. doi: 10.1186/s10020-025-01066-z (PMC11753094; doi:10.1186/s10020-025-01066-z)
Supplement: Supplementary file 1 — Additional file 1: Fig. S1. Related to Fig. 1B and 1C. Validation of 53BP1 depletion by si53BP1. Cells were transfected with siRNA against 53BP1 and incubated with bleomycin before harvest. Fig. S2. Related to Fig. 2. (A) Immunoblots for Fig. 2B and 2C. Cells were transfected with indicated siRNAs, and the lysates were subjected to immunoblotting. (B and C) Decreased recruitment of EGFP-53BP1 at DSBs induced by laser microirradiation in TLK knockout cells. The indicated U2OS cells were transfected with EGFP-53BP1 and irradiated with a 405 nm diode laser 24 hr post-transfection in the presence of BrdU. Live cell images (B) were captured every 5 seconds and data (C) represent the mean ± S.E.M. from eight cells. ***, P<0.001. (D and E) Decrease of 53BP1 focus formation in A549 TLK1 or TLK2 knockout cells. Representative images (D) and quantification of 53BP1 positive cells (E). Mean ± S.D. of triplicates. *, P < 0.05; **, P < 0.01: ****, P<0.0001. (F) Immunoblots for Fig. 2I. Fig. S3. Related to Fig. 4D and 4E. Immunoblots for Fig. 4D and 4E. Fig. S4. Related to Fig. 5. (A and B) Decrease of 53BP1 focus formation at DSBs in co-depletion of TLK1 and TLK2 in the absence of BRCA1. A549 cells were treated with cisplatin. Representative images (A) and quantitation (B). Scale bar, 10 μm. Mean ± S.D. of triplicates. ***, P < 0.005; ****, P < 0.0005. Fig. S5. Pan-cancer hazard analysis of low TLK1/2 cohort (Related to Fig. 6A). Cox coefficients of low TLK1/TLK2 cohorts were analyzed in multiple cancers. Red indicates higher risk (Poor outcome) and blue indicates lower risk (Good outcome) with respect to cohorts with low expression of TLK1 or TLK2. Samples are first divided by the mutation status of BRCA1, then further divided by the gene expression. ns, not significant. [file 10020_2025_1066_MOESM1_ESM.pdf]

Fig. S1_Kim et al.

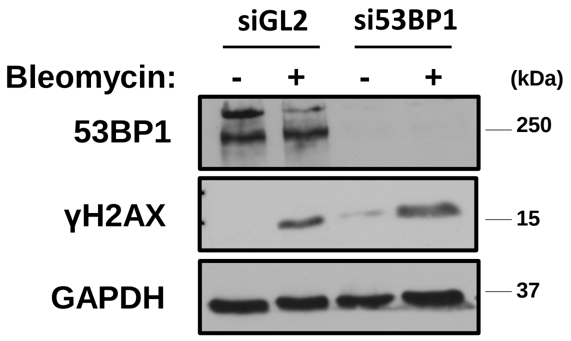

Fig. S1. Related to Fig. 1B and 1C. Validation of 53BP1 depletion by si53BP1. Cells were transfected with siRNA against 53BP1 and incubated with bleomycin before harvest.

Fig. S2_Kim et al.

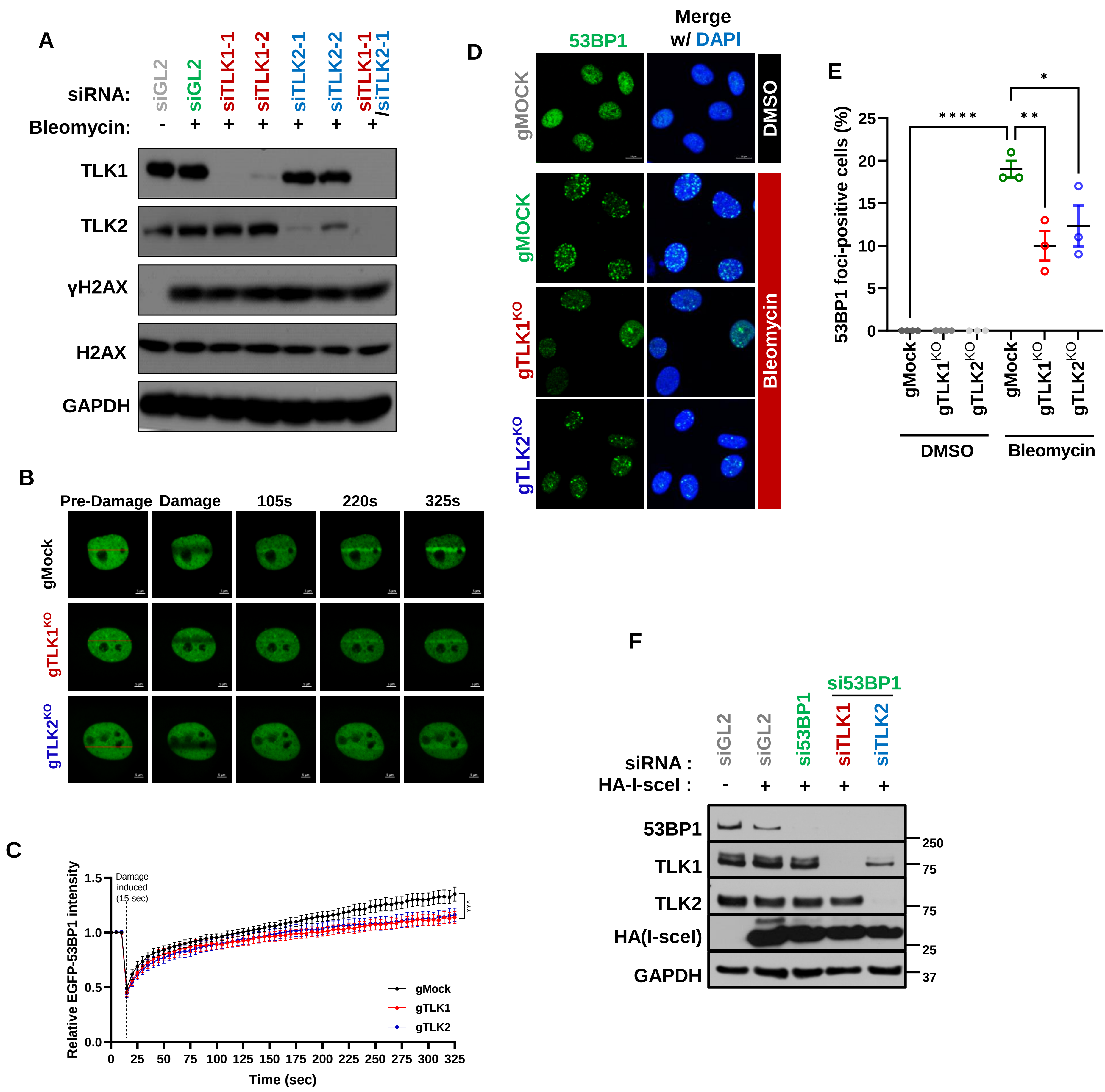

Fig. S2. Related to Fig. 2. (A) Immunoblots for Fig. 2B and 2C. Cells were transfected with indicated siRNAs, and the lysates were subjected to immunoblotting. . (B and C) Decreased recruitment of EGFP-53BP1 at DSBs induced by laser microirradiation in TLK knockout cells. The indicated U2OS cells were transfected with EGFP-53BP1 and irradiated with a 405 nm diode laser 24 hr post-transfection in the presence of BrdU. Live cell images (B) were captured every 5 seconds and data (C) represent the mean ± S.E.M. from eight cells. ***, P<0.001. (D and E) Decrease of 53BP1 focus formation in A549 TLK1 or TLK2 knockout cells. Representative images (D) and quantification of 53BP1 positive cells (E). Mean ± S.D. of triplicates. *, P < 0.05; **, P < 0.01: ****, P<0.0001. (F) Immunoblots for Fig. 2I. .

Fig. S3_Kim et al.

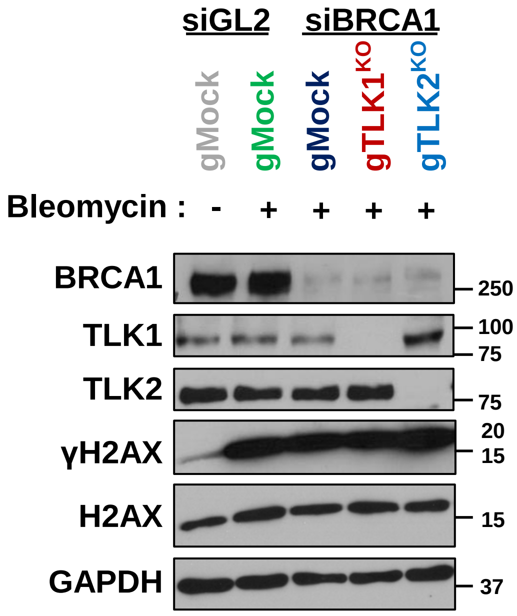

Fig. S3. Related to Fig. 4D and 4E. Immunoblots for Fig. 4D and 4E.

Fig. S4_Kim et al.

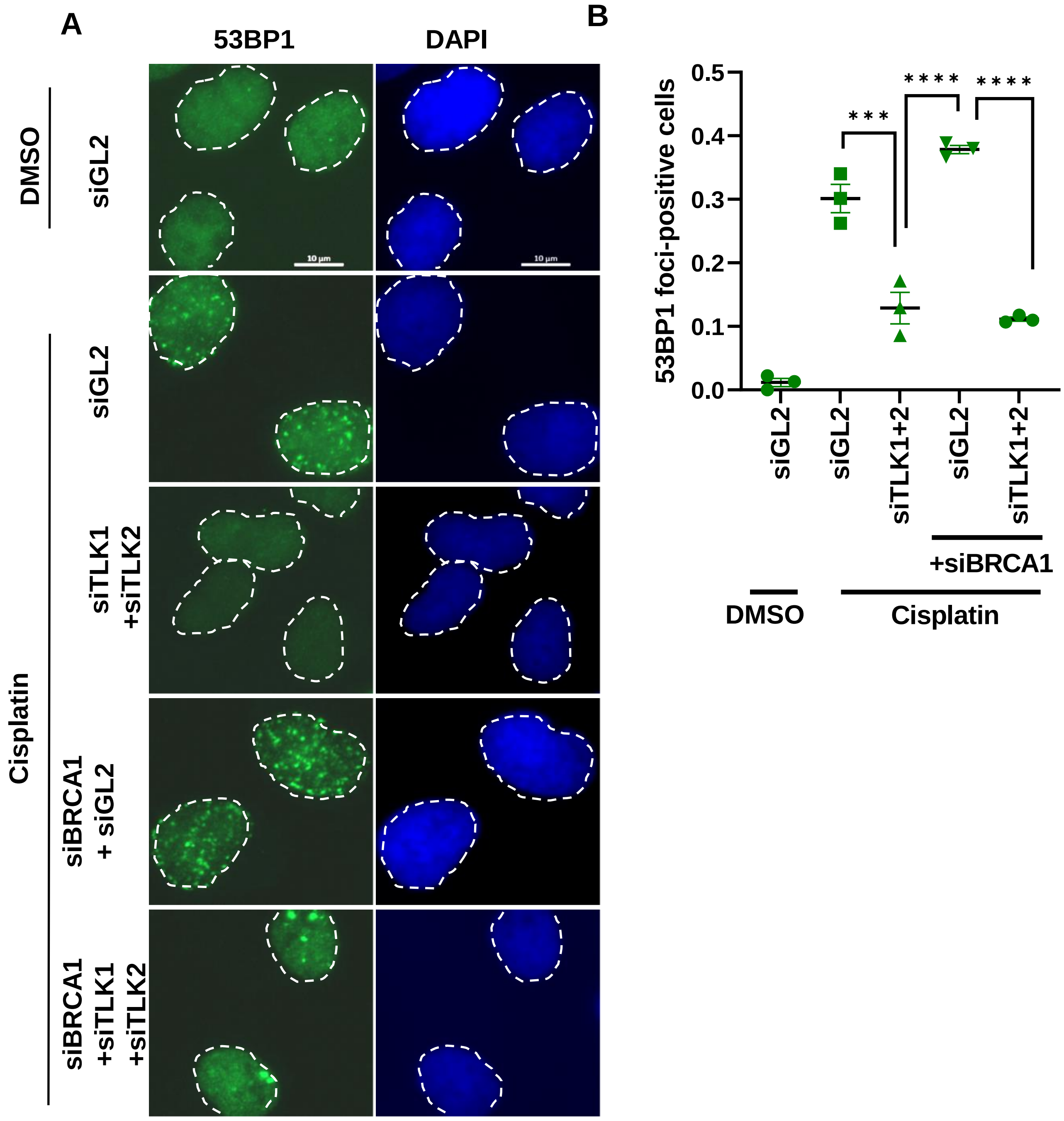

Fig. S4. Related to Fig. 5. **(**A and B) Decrease of 53BP1 focus formation at DSBs in co-depletion of TLK1 and TLK2 in the absence of BRCA1. A549 cells were treated with cisplatin. Representative images (A) and quantitation (B). Scale bar, 10 μm. Mean ± S.D. of triplicates. ***, $P < 0.005$; ****, $P < 0.0005$.

Fig. S5_Kim et al.

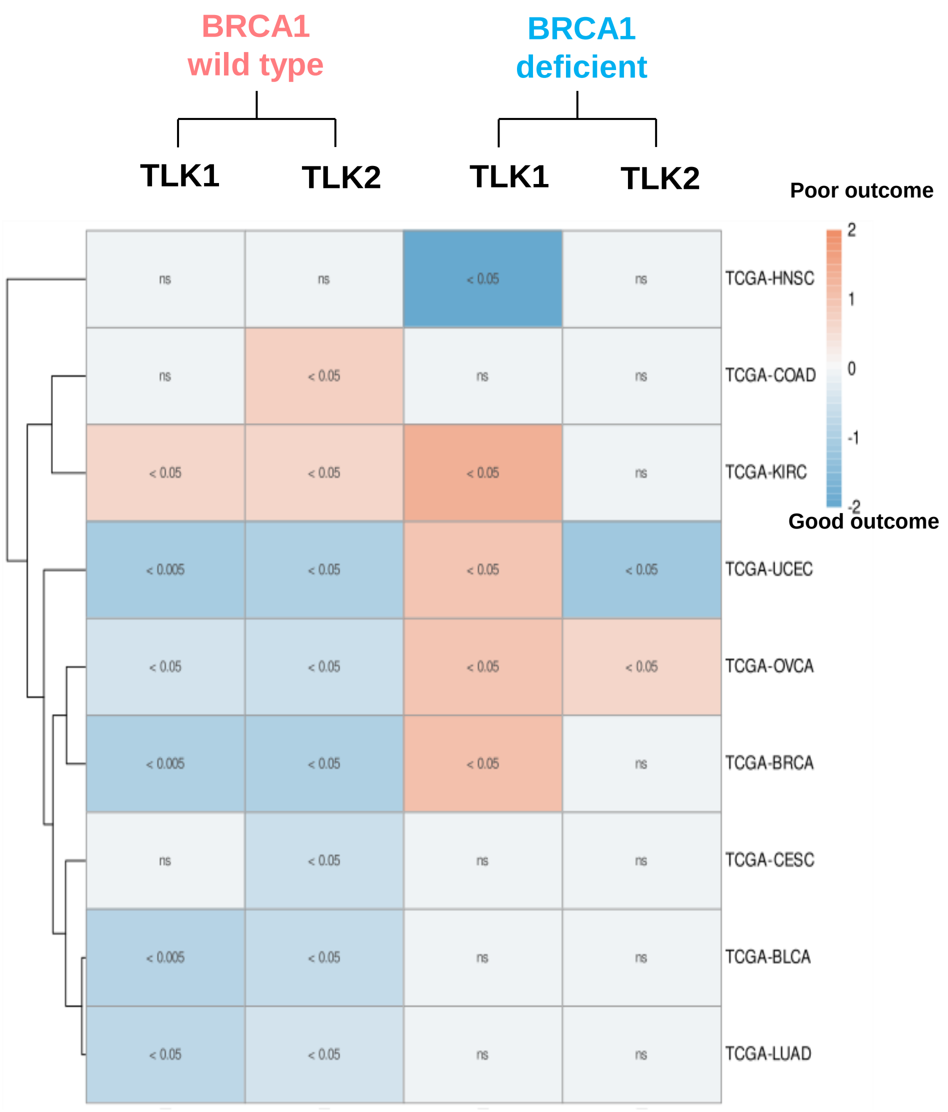

Fig. S5. Pan-cancer hazard analysis of low TLK1/2 cohort (Related to Fig. 6A). Cox coefficients of low TLK1/TLK2 cohorts were analyzed in multiple cancers. Red indicates higher risk (Poor outcome) and blue indicates lower risk (Good outcome) with respect to cohorts with low expression of TLK1 or TLK2. Samples are first divided by the mutation status of BRCA1, then further divided by the gene expression. ns, not significant.
